# Recombinant human activated protein C attenuates cardiovascular and microcirculatory dysfunction in acute lung injury and septic shock

**DOI:** 10.1186/cc9342

**Published:** 2010-11-26

**Authors:** Marc O Maybauer, Dirk M Maybauer, John F Fraser, Csaba Szabo, Martin Westphal, Levente Kiss, Eszter M Horvath, Yoshimitsu Nakano, David N Herndon, Lillian D Traber, Daniel L Traber

**Affiliations:** 1 Department of Anesthesiology, Investigational Intensive Care Unit, The University of Texas Medical Branch and Shriners Burns Hospital for Children, 301 University Blvd, Galveston, TX 77555-0591, USA; 2 Department of Anaesthesiology and Intensive Care, Philipps University of Marburg, Baldinger Str. 1, Marburg 35033, Germany; 3 Critical Care Research Group, University of Queensland and The Prince Charles Hospital at Brisbane, Rode Road, Chermside 4032, Queensland, Australia; 4 Institute of Human Physiology and Clinical Experimental Research, Semmelweis University, Tćzoltó utca 37-47, Budapest 1094, Hungary; 5 Department of Surgery, The University of Texas Medical Branch and Shriners Burns Hospital for Children, 815 Market Street, Galveston, TX 77550-2725, USA

## Abstract

**Introduction:**

This prospective, randomized, controlled, experimental animal study looks at the effects of recombinant human activated protein C (rhAPC) on global hemodynamics and microcirculation in ovine acute lung injury (ALI) and septic shock, resulting from smoke inhalation injury.

**Methods:**

Twenty-one sheep (37 ± 2 kg) were operatively prepared for chronic study and randomly allocated to either the sham, control, or rhAPC group (*n *= 7 each). The control and rhAPC groups were subjected to insufflation of four sets of 12 breaths of cotton smoke followed by instillation of live *Pseudomonas aeruginosa *into both lung lobes, according to an established protocol. Healthy sham animals were not subjected to the injury and received only four sets of 12 breaths of room air and instillation of the vehicle (normal saline). rhAPC (24 μg/kg/hour) was intravenously administered from 1 hour post injury until the end of the 24-hour experiment. Regional microvascular blood flow was analyzed using colored microspheres. All sheep were mechanically ventilated with 100% oxygen, and fluid resuscitated with lactated Ringer's solution to maintain hematocrit at baseline levels.

**Results:**

The rhAPC-associated reduction in heart malondialdehyde (MDA) and heart 3-nitrotyrosine (a reliable indicator of tissue injury) levels occurred parallel to a significant increase in mean arterial pressure and to a significant reduction in heart rate and cardiac output compared with untreated controls that showed a typical hypotensive, hyperdynamic response to the injury (*P *< 0.05). In addition, rhAPC significantly attenuated the changes in microvascular blood flow to the trachea, kidney, and spleen compared with untreated controls (*P *< 0.05 each). Blood flow to the ileum and pancreas, however, remained similar between groups. The cerebral blood flow as measured in cerebral cortex, cerebellum, thalamus, pons, and hypothalamus, was significantly increased in untreated controls, due to a loss of cerebral autoregulation in septic shock. rhAPC stabilized cerebral blood flow at baseline levels, as in the sham group.

**Conclusions:**

We conclude that rhAPC stabilized cardiovascular functions and attenuated the changes in visceral and cerebral microcirculation in sheep suffering from ALI and septic shock by reduction of cardiac MDA and 3-nitrotyrosine.

## Introduction

Every year, more than 750,000 patients in the United States develop sepsis, and 20 to 40% of these patients die [1]. The current understanding of the pathophysiology of sepsis is that inflammation, coagulation, and apoptosis are linked in the disease process [2]. Recombinant human activated protein C (rhAPC), a natural anticoagulant, is the first biological agent to have shown a significant survival benefit in patients with sepsis [3]. The protective effect of rhAPC in patients with severe sepsis is likely to reflect the ability of activated protein C (APC) to modulate multiple pathways. In addition to its anticoagulant properties, APC downregulates inflammatory and apoptotic responses [2].

Doubts about the beneficial protective effects of APC have persisted, however, and have been refueled by the recently published negative trials in less severely ill patients [4] and in children [5]. Infusion of rhAPC in human models of endotoxemia was also not shown to have any significant effect on proinflammatory responses or on thrombin generation [6,7].

Our group has recently shown that rhAPC improved pulmonary function in an ovine model of septic shock and pneumonia [8]. The improved oxygenation was based on a significant reduction of lung tissue 3-nitrotyrosine (3-NT), a reliable indicator of tissue injury caused by reactive nitrogen species such as peroxynitrite (ONOO^-^) [8]. Since it is known that ONOO^- ^formation is linked to the regulation of vascular tone [9], and that rhAPC has been shown to improve capillary perfusion from lipopolysaccharide-mediated microcirculatory dysfunction [10] and may attenuate intestinal ischemia/reperfusion-induced injury [11], we hypothesized that rhAPC administration likewise improves global hemodynamics and regional microvascular blood flow during septic shock.

On the basis of these observations and continuing controversy, it seems important to re-explore the effects of APC in relevant animal models. We therefore used a clinically relevant sepsis model to investigate whether APC could have beneficial therapeutic effects in septic shock.

## Materials and methods

The Institutional Animal Care and Use Committee of the University of Texas Medical Branch at Galveston approved the present study. The Investigational Intensive Care Unit at University of Texas Medical Branch is an Association for Assessment and Accreditation of Laboratory Animal Care International-approved facility. The guidelines of the National Institutes of Health for the care and use of experimental animals were carefully followed. The animals were individually housed in metabolic cages and were studied in the awake state.

### Experimental protocol

Twenty-one female Merino sheep (37 ± 2 kg) were included in the present study. For the operative procedures, sheep were anesthetized, and under aseptic conditions the animals were chronically instrumented for hemodynamic monitoring with a right femoral artery catheter, a 7-French Swan-Ganz™ thermodilution catheter, and a left atrial catheter, as previously described [12]. Following the surgical procedure, catheters were flushed with heparin, and the animals were allowed to recover for 7 days. During this time they had free access to food and water.

One day before the experiment commenced, catheters were connected to pressure transducers (Model PX3X3; Baxter Edwards Critical Care Division, Irvine, CA, USA) with continuous flushing devices. Electronically calculated mean pressures were recorded on a monitor with graphic and digital displays, and cardiac output (CO), core body temperature, arterial blood gases, and carboxyhemoglobin (COHb) saturation were measured as reported [13]. The cardiac index and the systemic vascular resistance index were calculated using standard equations [14]. Protein concentrations in plasma were measured with a refractometer.

Following a baseline (BL) measurement, sheep were randomly allocated to one of three groups (*n *= 7 each): an uninjured, untreated sham group; an injured, untreated control group; and an injured group treated with rhAPC. A tracheostomy was performed under ketamine anesthesia (10 mg/kg), and a Foley urinary retention catheter was placed in all animals to measure urine output. Anesthesia was then maintained using 1.5 to 2.5% halothane (Vedco Inc., St Joseph, MO, USA) in oxygen. The animals allocated to the control and treatment groups were subjected to smoke inhalation injury (four sets of 12 breaths of cotton smoke, <40°C), according to an established protocol [12]. The sham group received four sets of 12 breaths of room air. Arterial COHb plasma concentrations were determined after each set of smoke or air inhalation and served as an index of lung injury. After smoke inhalation, an experimental bacterial solution (live *Pseudomonas aeruginosa*, 5 × 10^11 ^colony-forming units) was instilled into the lungs of control and treatment animals using a bronchoscope (Model PF-P40; Olympus America Inc. Melville, NY, USA). The sham group received only the vehicle (NaCl 0.9%). Anesthesia was then discontinued and the sheep were allowed to awaken [12]. In the treatment group, rhAPC was intravenously administered, using the clinically established dose of 24 μg/kg/hour [3,8], which also has been shown to be adequate in sheep [15,16]. The control group received only the vehicle (NaCl 0.9%). Both infusions were initiated 1 hour post injury, and lasted until the end of the experiment.

All animals were mechanically ventilated (Servo-Ventilator 900C; Siemens, Elema, Sweden) with a FiO_2 _of 1.0, an initial tidal volume of 15 ml/kg and a respiration rate of 30/minute. For the duration of the 24-hour study period, ventilator settings were periodically adjusted to maintain an arterial pressure of carbon dioxide (pCO_2_) below BL values because this approach allows invasive ventilation in sheep in the awake state. The ventilatory settings were adapted to the physiology of the sheep. Since the lungs of sheep have a higher compliance than those of the human, a tidal volume of 15 ml/kg body weight was used to prevent atelectasis. Such volumes result in peak and plateau pressures of approximately 20 mmHg and are similar to a 6 to 8 to 10 ml/kg tidal volume in humans, depending on individual lung compliance. Positive end-expiratory pressure remained at a fixed level of 6 cmH_2_O to avoid ventilation-related differences in the study groups. These ventilator settings were chosen in accordance with those originally described for this model by Murakami and colleagues [12].

All animals were fluid resuscitated, initially started with an infusion rate of 2 ml/kg/hour lactated Ringer's solution. The infusion rate was then adjusted to maintain hematocrit at BL levels. During the 24-hour study period, all animals had free access to food, but not to water, to precisely control the fluid balance.

### Measurement of plasma nitrate/nitrite formation

The concentration of total amount of nitric oxide metabolites (NO_x_) in the plasma was measured intermittently by a blinded co-investigator. Plasma samples were subjected to NO_x _reduction using vanadium(III) as a reducing agent in a commercial instrument (model 745; Antek Instruments, Houston, TX, USA). The resulting nitric oxide (NO) was measured with a chemiluminescent NO analyzer (model 7020; Antek) and was recorded by dedicated software as the NO content (in μM) [12].

### Regional microvascular blood flow measurements

The determination of regional blood flow was performed using colored microspheres. Approximately 5 million microspheres (15.0 ± 0.1 μm) were injected into the left atrium at BL, 6, 12, and 24 hours, while reference blood was withdrawn from the femoral arterial catheter at a constant rate of 10 ml/minute. The color of the microspheres was randomized for each injection. During necropsy, representative transmural tissue samples were obtained from the distal trachea, pancreas, spleen, both kidneys (cortex), and ileum. In addition, brain tissue samples of the cerebral cortex, cerebellum, thalamus, pons, and hypothalamus were obtained. All these tissue samples were analyzed by Interactive Medical Technologies Ltd (Los Angeles, CA, USA) by determining the weight of each tissue sample, digesting the entire sample in a high concentration of NaOH, and measuring the total number of different colored spheres using flow cytometry. Regional blood flow was then calculated using the following formula [14,17]:

$$\begin{array}{c} \text{Regional}\;\text{blood}\;\text{flow}\;(\text{ml}/\text{minute}/\text{g})=(\text{total}\;\text{tissue}\;\text{spheres})/(\text{tissue}\;\text{weight},\;\text{g})\times \\ \left(\text{reference}\;\text{spheres}/\text{ml}/\text{minute})\right) \end{array}$$

### Necropsy

After completion of the experiment, the animals were anesthetized with ketamine (15 mg/kg) and sacrificed by intravenous injection of 60 ml saturated potassium chloride. Immediately after death, heart tissue was excised for determination of heart 3-NT, and heart malondialdehyde (MDA) as described below [18].

### Quantification of malondialdehyde activity

MDA is a major end-product of oxidation of polyunsaturated fatty acids, and is frequently measured as an indicator of lipid peroxidation and oxidative stress. Using a commercially available kit, heart tissue was homogenized (100 mg/ml) in 1.15% KCl buffer. To the tissue homogenate, 20% trichloroacetic acid, 0.67% thiobarbituric acid, and 2% butylated hydroxytoluene were added, and the mixture was incubated for 30 minutes at 95°C. After cooling to room temperature, *n*-butanol was also added and shaken vigorously. After centrifugation at 2,500 × *g *for 10 minutes, the organic layer was taken and its absorbance at 532 nm was measured. 1,1,3,3-Tetramethoxypropane was used as an external standard [18].

### ELISA for heart 3-nitrotyrosine

Quantification of heart tissue 3-NT content was analyzed using ELISA as previously described [18]. Briefly, 2 ml of 10× diluted homogenation buffer (1:10; Cayman Chemical, Ann Arbor, MI, USA) containing 250 mM Tris-HCl (pH 7.4), 10 mM ethylenediamine tetraacetic acid and 10 mM ethyleneglycol-bis(β-aminoethylether)-*N*,*N*,*N*',*N*'-tetraacetic acid were added to 200 mg freshly frozen heart tissue and then homogenized. Following centrifugation (10,000 × *g *at 4°C) for 15 minutes, 100 μg supernatant was used for assessment of 3-NT. Measurements were performed using the HyCult biotechnology 3-NT solid-phase ELISA (Cell Sciences Inc, Canton, MA, USA), and were strictly performed according to the manufacturer's protocol.

Following incubation for 1 hour, the plate was emptied and washed three times (20 seconds each) using 200 μl wash buffer provided with the kit. Thereafter, 100 μl diluted tracer were added to each well and incubated for 1 hour. Following the washing process, 100 μl diluted streptavidin-peroxidase conjugate was added and incubated for an additional hour. After having repeated the washing procedure, 100 μl freshly prepared tetramethylbenzidine were added and incubated for 25 minutes. The reaction was then stopped by adding 100 μl stop solution to the samples. Finally, the tray was placed in a spectrophotometer and the absorbance determined at a wavelength of 450 nm, following the instructions provided by the manufacturer.

### Statistical analysis

For statistical analysis, Sigma Stat 2.03 software (SPSS Inc., Chicago, IL, USA) was used. After confirming a normal distribution (Kolmogorov-Smirnov test), a two-way analysis of variance for repeated measurements with appropriate Student-Newman-Keuls *post hoc *comparisons was used to detect differences within and between groups. Significance was assumed when *P *< 0.05. Data are presented as means ± standard errors of the mean.

## Results

### Injury and survival

The arterial COHb determined immediately after the fourth set of smoke exposure averaged 73 ± 5% in the control group and 70 ± 4% in the rhAPC group. The sham group, which was not exposed to smoke inhalation, showed a COHb level of 5 ± 1% after giving four sets of 12 breaths of room air. No significant difference was determined in COHb levels (*P *> 0.05) for the injured groups, reflecting the consistency of the injury. With aggressive fluid challenge, all animals survived the 24-hour study period.

### Global hemodynamics

**Figure 1:**
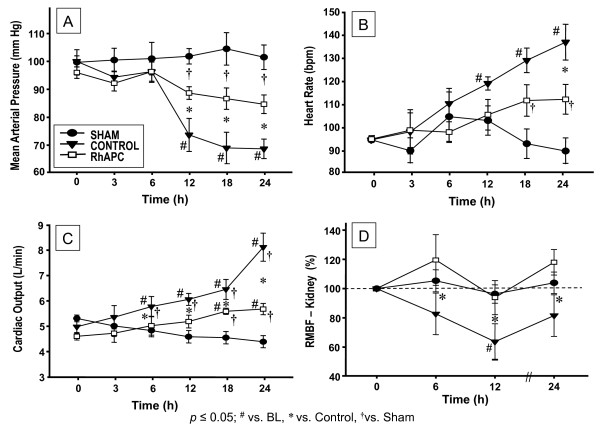
**Changes in global hemodynamics**. Changes in **(a) **mean arterial pressure (mmHg), **(b) **heart rate (bpm), **(c) **cardiac output (l/minute), and **(d) **regional microvascular blood flow (RMBF) in kidney cortex (percentage of baseline). Data expressed as mean ± standard error of the mean of seven animals per group. Significance *P *< 0.05: #versus baseline (BL = 0 hours); †versus sham; *versus control. rhAPC, recombinant human activated protein C.

Cardiovascular variables were stable in sham animals. In the control group, the heart rate and CO increased significantly after 24 hours and were associated with a significant drop in mean arterial pressure (MAP) (Figure 1 each *P *< 0.05 vs. BL). In rhAPC-treated sheep, the CO and heart rate remained stable, and MAP did not fall to the same extent as in control sheep (each *P *< 0.05). Global hemodynamic data are presented in Table 1.

**Table 1:** Global hemodynamics

Parameter	Group	BL = 0 hours	3 hours	6 hours	12 hours	18 hours	24 hours
CVP (mmHg)	Sham	7 ± 1	13 ± 2^#^	12 ± 1^#^	11 ± 1^#^	10 ± 1^#^	10 ± 1^#^
	Control	6 ± 1	9 ± 1^#^	11 ± 1^#^	13 ± 1^#^	14 ± 1^#**†**^	15 ± 1^#**†**^
	rhAPC	5 ± 1	10 ± 1^#^	11 ± 1^#^	11 ± 1^#^	13 ± 1^#^	13 ± 2^#^

MPAP (mmHg)	Sham	23 ± 1	26 ± 1	27 ± 1	26 ± 1	26 ± 1	27 ± 1^#^
	Control	21 ± 1	26 ± 2^#^	31 ± 2^#**†**^	28 ± 1^#^	30 ± 1^#^	33 ± 1^#**†**^
	rhAPC	20 ± 1	25 ± 1^#^	25 ± 1^#^*	27 ± 2^#^	30 ± 2^#^	28 ± 2^#^*

PAOP (mmHg)	Sham	11 ± 1	14 ± 1^#^	15 ± 1^#^	14 ± 1^#^	16 ± 1^#^	14 ± 1^#^
	Control	10 ± 1	15 ± 1^#^	16 ± 1^#^	17 ± 1^#^	17 ± 1^#^	18 ± 1^#**†**^
	rhAPC	11 ± 1	16 ± 1^#^	16 ± 1^#^	17 ± 1^#^	17 ± 1^#^	16 ± 1^#^*

LAP (mmHg)	Sham	7 ± 0	9 ± 1^#^	10 ± 1^#^	9 ± 1^#^	9 ± 1^#^	9 ± 1^#^
	Control	7 ± 1	11 ± 1^#^	15 ± 2^#**†**^	15 ± 1^#**†**^	17 ± 1**^†^**	19 ± 1^#**†**^
	rhAPC	9 ± 2	9 ± 1	11 ± 1*	13 ± 1^#**†**^	15 ± 1^#**†**^	14 ± 2^#†^*

CI (l/min/m^2^)	Sham	5.6 ± 0.1	5.3 ± 0.3	5.1 ± 0.1	4.8 ± 0.1	4.8 ± 0.1	4.6 ± 0.1
	Control	5.3 ± 0.3	5.8 ± 0.5	6.2 ± 0.4^#**†**^	6.5 ± 0.3^#**†**^	6.9 ± 0.3^#**†**^	8.7 ± 0.4^#**†**^
	rhAPC	5.0 ± 0.2	5.2 ± 0.4	5.5 ± 0.4	5.7 ± 0.3^†^*	6.1 ± 0.2^#†^*	6.2 ± 0.2^#†^*

SVRI (dyne s/cm^5^/m^2^)	Sham	1289 ± 87	1374 ± 133	1402 ± 103	1502 ± 64	1576 ± 79	1599 ± 96
	Control	1419 ± 59	1224 ± 92	1128 ± 85^#^	740 ± 50^#**†**^	632 ± 52^#**†**^	502 ± 42^#**†**^
	rhAPC	1464 ± 79	1364 ± 187	1334 ± 188	1111 ± 64^#†^*	968 ± 51^#†^*	934 ± 41^#†^*

PaO_2_:FiO_2 _ratio	Sham	506 ± 10	471 ± 11	490 ± 18	492 ± 17	498 ± 16	491 ± 19
	Control	521 ± 19	202 ± 36^#**†**^	144 ± 26^#**†**^	72 ± 5^#**†**^	72 ± 7^#**†**^	74 ± 6^#**†**^
	rhAPC	541 ± 10	245 ± 56^#**†**^	176 ± 39^#**†**^	151 ± 24^#†^*	134 ± 22^#†^*	118 ± 17^#**†**^

PaCO_2 _(mmHg)	Sham	32 ± 1	29 ± 1	26 ± 1^#^	25 ± 1^#^	23 ± 2^#^	24 ± 1^#^
	Control	37 ± 2	29 ± 3^#^	27 ± 3^#^	28 ± 2^#^	29 ± 0^#^	29 ± 2^#^
	rhAPC	36 ± 0	25 ± 3^#^	27 ± 2^#^	25 ± 2^#^	27 ± 2^#^	27 ± 4^#^

apH (-log_10_[H^+^])	Sham	7.469 ± 0.012	7.542 ± 0.027^#^	7.537 ± 0.014^#^	7.558 ± 0.019^#^	7.583 ± 0.015^#^	7.548 ± 0.026^#^
	Control	7.449 ± 0.014	7.577 ± 0.031^#^	7.564 ± 0.025^#^	7.525 ± 0.012^#^	7.465 ± 0.031**^†^**	7.437 ± 0.027^†^
	rhAPC	7.470 ± 0.010	7.622 ± 0.026^#**†**^	7.590 ± 0.018^#^	7.556 ± 0.016^#^	7.509 ± 0.024^†^	7.480 ± 0.043^†^

DO_2_I (ml/min/m^2^)	Sham	607 ± 33	651 ± 49	648 ± 57	567 ± 48	565 ± 38	551 ± 41
	Control	580 ± 70	663 ± 58	719 ± 62#	709 ± 27^#^	726 ± 67^#^	984 ± 82^#**†**^
	rhAPC	624 ± 32	638 ± 43	690 ± 51	644 ± 56	650 ± 40	666 ± 37^#^*

VO_2_I (ml/min/m^2^)	Sham	209 ± 21	198 ± 26	183 ± 21	170 ± 21	195 ± 31	165 ± 23
	Control	242 ± 24	220 ± 30	231 ± 30	215 ± 35	178 ± 29	274 ± 41
	rhAPC	197 ± 16	244 ± 24	227 ± 24	193 ± 31	186 ± 25	253 ± 39

Temperature (°C)	Sham	39.0 ± 0.1	39.5 ± 0.1	39.5 ± 0.1	39.2 ± 0.2	39.1 ± 0.1	39.2 ± 0.1
	Control	39.1 ± 0.1	40.2 ± 0.2^#**†**^	40.5 ± 0.2^#**†**^	40.4 ± 0.2^#**†**^	40.1 ± 0.2^#**†**^	40.1 ± 0.3^#**†**^
	rhAPC	39.3 ± 0.1	40.0 ± 0.2^#**†**^	40.5 ± 0.2^#**†**^	40.5 ± 0.1^#**†**^	40.4 ± 0.2^#**†**^	40.4 ± 0.1^#**†**^

Hematocrit (%)	Sham	25.2 ± 0.8	26.2 ± 1.2	26.0 ± 1.6	25.0 ± 1.3	24.7 ± 1.0	24.7 ± 1.0
	Control	25.3 ± 1.0	27.5 ± 0.9^#^	28.3 ± 0.8^#^	29.7 ± 0.9^#**†**^	28.8 ± 1.2^#^	28.9 ± 0.7^#**†**^
	rhAPC	28.2 ± 0.9	27.7 ± 1.1	29.7 ± 1.3	28.3 ± 0.9^†^	28.3 ± 1.0	27.2 ± 0.7^†^

Plasma NO_x _(μM)	Sham	5.0 ± 0.6	5.4 ± 0.6	5.4 ± 0.8	5.0 ± 0.9	5.8 ± 0.7	6.0 ± 0.7
	Control	5.2 ± 0.5	7.1 ± 0.8^#^	7.9 ± 0.8^#**†**^	9.0 ± 1.2^#**†**^	9.8 ± 1.1^#**†**^	10.5 ± 1.1^#**†**^
	rhAPC	4.6 ± 0.6	7.8 ± 0.6^#^	9.4 ± 0.5^#**†**^	9.6 ± 1.1^#**†**^	9.5 ± 1.1^#**†**^	8.9 ± 0.8^#**†**^

Plasma Onc (mmHg)	Sham	21.9 ± 0.4	20.9 ± 0.7	20.2 ± 0.9	20.8 ± 0.6	22.1 ± 0.5	22.5 ± 0.7
	Control	22.7 ± 0.9	19.5 ± 0.4^#^	16.2 ± 0.7^#**†**^	14.4 ± 0.7^#**†**^	12.5 ± 0.8^#**†**^	10.9 ± 1.0^#**†**^
	rhAPC	24.3 ± 0.4	22.7 ± 0.3*	21.0 ± 0.3^#^*	18.3 ± 0.7^#†^*	15.9 ± 1.3^#†^*	14.7 ± 1.5^#†^*

CVP, central venous pressure; MPAP, mean pulmonary artery pressure; PAOP, pulmonary artery occlusion pressure; LAP, left atrial pressure; CI, cardiac index; SVRI, systemic vascular resistance index; PaO_2_:FiO_2 _ratio, Horovitz quotient; PaCO_2_, arterial carbon dioxide partial pressure; apH, arterial pH; DO_2_I, oxygen delivery index; VO_2_I, oxygen consumption index; NOx, nitrate-to-nitrite formation; Onc, oncotic pressure; rhAPC, recombinant human activated protein C. *P *≤0.05: ^#^versus baseline, ^†^versus sham, *versus control.

### Regional microvascular blood flow

**Figure 2:**
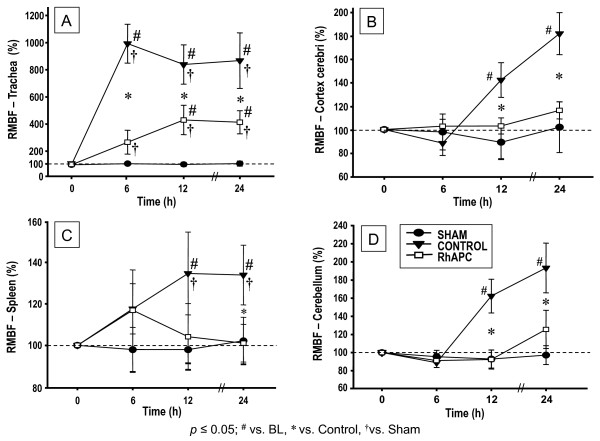
**Regional microvascular blood flow**. Microvascular blood flow (RMBF) in the **(a) **trachea, **(b) **cerebral cortex, **(c) **spleen, and **(d) **cerebellum (percentage of baseline). Data expressed as mean ± standard error of the mean of seven animals per group. Significance *P *< 0.05: #versus baseline (BL = 0 hours); †versus sham; *versus control. rhAPC, recombinant human activated protein C.

The regional microvascular blood flow in all sham animals remained near BL levels and showed no statistical difference to BL. In the trachea, below the tracheostomy tube, blood flow dramatically increased in control animals during the entire experiment versus BL, versus sham, and versus rhAPC-treated sheep (Figure 2* P *< 0.05). In addition, the regional microvascular blood flow of control animals in both kidneys as well as in the pancreas significantly decreased over time versus BL and versus sham animals (*P *< 0.05 each). Pancreatic blood flow in the control group was lower, but was not statistically different from the rhAPC group (Table 2). Blood flow in both kidneys, however, did not fall to the same extent in rhAPC-treated sheep and was significantly attenuated over time (*P *< 0.05, Figure 1). In the spleen, blood flow significantly increased in controls compared with BL, sham, and rhAPC groups (*P *< 0.05, Figure 2). The regional microvascular blood flow in the ileum significantly increased in controls, compared with BL and sham animals (*P *< 0.05, Table 2), but was not statistically different compared with the rhAPC group.

**Table 2:** Regional microvascular blood flow

Parameter	Group	BL = 0 hours	6 hours	12 hours	24 hours
Cardiac index (l/min/m^2^)	Sham	100 ± 0	90.7 ± 2.8	86.2 ± 3.0	82.5 ± 3.0
	Control	100 ± 0	117.1 ± 10.0	122.7 ± 5.9^#†^	166.2 ± 16.2^#†^
	rhAPC	100 ± 0	109.6 ± 9.2	113.2 ± 6.6*^†^	124.2 ± 6.9*^†^

Pancreas (%)	Sham	100 ± 0	97.3 ± 10.5	94.9 ± 8.1	104.0 ± 16.1
	Control	100 ± 0	55.5 ± 9.3	60.5 ± 12.3^#^	52.3 ± 6.9^#†^
	rhAPC	100 ± 0	76.5 ± 9.5	61.3 ± 9.5^#^	65.5 ± 15.2^#^

Ileum (%)	Sham	100 ± 0	95.4 ± 20.2	89.1 ± 11.3	111.8 ± 23.5
	Control	100 ± 0	102.4 ± 13.5	156.0 ± 38.6^#†^	135.1 ± 28.1
	rhAPC	100 ± 0	95.6 ± 9.2	107.8 ± 19.0	136.0 ± 32.3

Medulla oblongata (%)	Sham	100 ± 0	91.3 ± 11.2	95.5 ± 8.3	93.1 ± 17.1
	Control	100 ± 0	78.1 ± 7.1	132.1 ± 17.5	145.0 ± 16.7^#^
	rhAPC	100 ± 0	75.3 ± 9.3	89.6 ± 22.4	97.2 ± 16.9

Thalamus (%)	Sham	100 ± 0	92.6 ± 7.4	95.2 ± 9.9	93.9 ± 13.8
	Control	100 ± 0	90.2 ± 9.3	137.0 ± 18.1^#^	157.7 ± 31.6^#^
	rhAPC	100 ± 0	96.1 ± 4.8	95.8 ± 9.1*	107.5 ± 10.3*

Pons (%)	Sham	100 ± 0	95.9 ± 9.9	92.6 ± 7.5	96.1 ± 18.3
	Control	100 ± 0	87.7 ± 8.8	130.0 ± 14.7^#^	167.3 ± 26.7^#†^
	rhAPC	100 ± 0	78.1 ± 6.0	81.8 ± 9.1*	104.4 ± 15.5*

Regional microvascular blood flow (percentage from baseline). rhAPC, recombinant human activated protein C. *P *≤0.05: ^#^vs. BL, *vs. control, ^†^vs. sham.

The cerebral blood flow was measured in the cerebral cortex, cerebellum, thalamus, pons, and hypothalamus. In all these areas of control animals, cerebral blood flow was significantly increased compared with BL, sham, and rhAPC animals (*P *< 0.05, Figure 2 and Table 2). There was no statistical difference between sham and rhAPC.

### Plasma nitrate/nitrite levels

Plasma NO_x _levels increased significantly over time in the control and rhAPC groups, as compared with the sham group (*P *< 0.05). There was no statistical difference between the two injured groups (Table 1).

### Plasma oncotic pressure

The plasma oncotic pressure was significantly decreased in both injured groups versus BL and versus the sham group, which remained at BL levels (*P *< 0.05). The reduction in plasma oncotic pressure was significantly attenuated in the rhAPC group as compared with the control group (*P *< 0.05, Table 1).

### Pulmonary function

The pulmonary variables showed similar results as previously described, and are presented in Table 1.

### Tissue analysis

**Figure 3:**
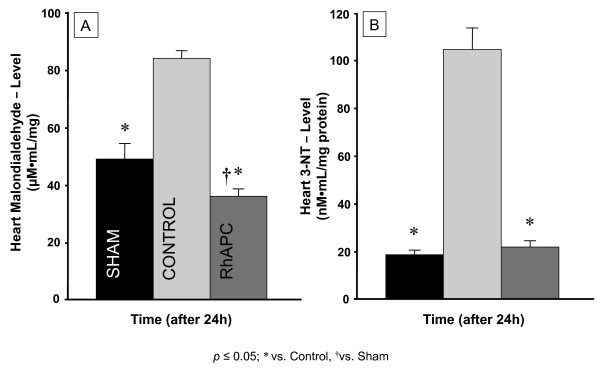
**Tissue analysis**. Levels of **(a) **heart malondialdehyde (MDA, μM/ml/mg) and **(b) **heart 3-nitrotyrosine (3-NT, nM/ml/mg protein). Data expressed as mean ± standard error of the mean of seven animals per group. Significance *P *< 0.05: †versus sham; *versus control. rhAPC, recombinant human activated protein C.

The results for heart 3-NT are shown in Figure 3. The control group showed a significantly higher protein concentration than the sham group (*P *< 0.05). The concentration in the rhAPC group showed no statistical difference to the sham group, but was significantly lower (*P *< 0.05) than in the control group.

Heart MDA levels (μM/ml/mg) of controls were significantly higher than sham or rhAPC levels (*P *< 0.05, Figure 3). There was no statistical difference between sham and rhAPC animals.

### Total fluid balance

Over 24 hours, the total urine output in sham animals (3,459 ± plusorminus 289 ml) was significantly higher than in control animals (1,353 ± plusorminus 260 ml) and rhAPC animals (2,049 ± plusorminus 170 ml, *P *< 0.05 each). Urine output in rhAPC-treated animals was significantly higher than in controls (*P *< 0.05).

The sham group received a total of 1,832 ± plusorminus 119 ml fluids. This fluid intake was significantly less than in controls (3,534 ± plusorminus 529 ml) or rhAPC animals (5,019 ± plusorminus 1,091 ml; *P *< 0.05 each). The fluid intake in the rhAPC group was significantly greater than in controls (*P *< 0.05).

The total fluid balance reflects the total urine output, subtracted from the total fluid intake over 24 hours, when started with a rate of 2 ml/kg/hour. The control group (2,181 ± 577 ml) and the rhAPC group (2,970 ± 1,076 ml) both had significantly greater positive fluid balances than the sham group (-1,627 ± 227 ml, *P *< 0.05 each). There was no difference between the injured groups.

### Temperature

Core body temperature remained at baseline in the sham group. The control and rhAPC group showed a significant increase in temperature as compared with the sham group and versus BL (*P *< 0.05). There was no statistical difference between the injured groups (Table 1).

## Discussion

The present study investigated the effects of rhAPC on global hemodynamics and regional microvascular blood flow in an established and clinically relevant model of septic shock resulting from smoke inhalation injury [8,12-14]. The major finding was a significantly improved cardiovascular function by rhAPC treatment, indexed by stabilized MAP, heart rate, and CO as well as attenuated changes in visceral and cerebral microcirculation to certain organs. Whereas blood flow of the ileum and pancreas remained unchanged between the injured groups, the changes in blood flow to the renal cortex, spleen, trachea, cerebral cortex, cerebellum, thalamus, pons, and hypothalamus were attenuated in the rhAPC-treated group.

The sheep model of acute lung injury (ALI) and septic shock is suitable for studying the effects of sepsis, because it closely mimics the pathophysiology of human sepsis [12]. This two-hit model fulfills the criteria of sepsis as described by Bone and colleagues [19], and would lead to decreased regional microvascular blood flow to most, if not all, vital organs - thereby mimicking the anticipated mechanisms for the development of multiorgan dysfunction syndrome [14].

Our group has recently shown that rhAPC improved pulmonary function in this ovine model by reduction of airway obstruction and lung tissue 3-NT levels, a reliable indicator of tissue injury caused by reactive nitrogen species such as ONOO^- ^[8]. In the latter study, the activated clotting time and platelet count remained stable in rhAPC-treated animals. In addition, rhAPC prevented disseminated intravascular coagulation.

Among the various anticoagulants, rhAPC is an especially important compound as it has shown a significant survival benefit in patients with severe sepsis [3]. The positive effects of rhAPC on pulmonary function in different models of ALI are well described [8,15,16,20-22]. The nonpulmonary, systemic effects of ALI, however, remain to be investigated.

Kalil and colleagues have shown that rhAPC contributed to an increase in MAP after endotoxin exposure of volunteers [6], and Monnet and colleagues reported that APC administration required less norepinephrine to maintain arterial blood pressure [23]. Wang and colleagues demonstrated beneficial cardiopulmonary effects of rhAPC in an ewe model of sepsis caused by peritonitis [24]. The pulmonary effects were comparable with our previous findings [8]. The exact mechanisms of the improved hemodynamic effects of rhAPC, however, are still not well defined. Hauser and colleagues recently described that overproduction of NO by inducible nitric oxide synthase is critically involved in the pathogenesis of circulatory shock [25]. Not only NO itself, via cyclic guanosine monophosphate-mediated smooth muscle relaxation, but also its downstream biological effects may play a role in arterial hypotension [26]. ONOO^- ^is a highly toxic reactive species formed from NO and superoxide (O_2_^-^), and is capable of inducing endothelial dysfunction and vascular hyporeactivity [27]. Recent data showed the implication of ONOO^- ^in the inactivation of α_1_-adrenoreceptors [28] and norepinephrine [29], and showed that superoxide deactivates catecholamines, resulting in loss of their vasopressor activity, consecutively resulting in hypotension [30]. Since we have previously shown significantly reduced pulmonary 3-NT levels of rhAPC-treated animals compared with controls in this model [8], we hypothesized that there is a link between rhAPC, ONOO^- ^and vascular regulatory mechanisms. The data of our present study clearly show less cardiac 3-NT formation, indicating less production of reactive nitrogen species such as ONOO^- ^following rhAPC infusion in ALI and septic shock. This, in turn, was associated with improved vascular tone and improved MAP as well as the systemic vascular resistance index. The attenuation of changes in organ perfusion necessitated less compensatory increase of the heart rate and CO.

In this context, it is well known that the interaction between leukocytes and endothelial cells is critical in endothelial cell damage. In our study there was no difference in NO_x _levels between the injured groups. This finding stands in contrast to the findings of Isobe and colleagues, who reported that APC prevented endotoxin-induced hypotension in rats by the inhibition of NO [31]. This contradiction might be related to the different species used, as well as to the timing of treatment in different animal models [20]. In our study, it is most probable that the prevention of 3-NT formation resulted from a reduction in oxidative stress as indicated by significantly reduced cardiac MDA levels. Sturn and colleagues demonstrated that neutrophils express receptors for APC, and also that neutrophil chemotaxis is inhibited by exposure to protein C, APC, or rhAPC [32]. APC can improve the visceral microcirculation by attenuating leukocyte-endothelial interactions and leukocyte rolling [33].

Importantly, Marechal and colleagues have shown that the endothelial glycocalyx is extremely sensitive to free radicals [34]. Oxidative stress was evaluated by oxidation of dihydrorhodamine in microvascular beds and levels of heart MDA and plasma carbonyl proteins, which were all increased in lipopolysaccharide-treated rats. APC enhanced the systemic arterial pressure response to norepinephrine in lipopolysaccharide-treated rats, and prevented capillary perfusion deficit in the septic microvasculature that was associated with reduced oxidative stress and preservation of the glycocalyx. It is obvious that lipopolysaccharide-induced major microcirculation dysfunction accompanied by microvascular oxidative stress and glycocalyx degradation may be limited by APC. This is in line with our findings, clearly showing that reduction in cardiac MDA and 3-NT led to attenuated changes in microvascular blood flow to eight out of 10 investigated organs. In our study, the attenuated drop in renal blood flow in rhAPC-treated animals, resulting from decreased MDA and 3-NT levels, is also in accordance with the findings of Gupta and colleagues, who demonstrated that administration of APC improved systemic hemodynamics and protected from renal dysfunction [35]. The antithrombotic properties [8] and cytoprotective properties [35] of APC further contribute to improved organ blood flow. The dramatic increase of tracheal blood flow in the present study was anticipated, given the degree of direct inflammatory damage by smoke inhalation at this site [14,17]; however, the significant decrease in tracheal blood flow of rhAPC-treated animals may be a direct anti-inflammatory effect. Blood flow to the ileum increased continuously in control animals, but rhAPC-treated sheep showed a significantly lower ileal blood flow at 12 hours post injury than controls. This might be considered a disadvantage of the rhAPC treatment, since restricted gut perfusion is known to result in bacterial translocation. The importance of this finding may remain controversial, however, because the blood flow in rhAPC-treated sheep was statistically not different from that of healthy sham animals.

In respect of cerebral blood flow, it is noteworthy that all animals in our study were moderately hyperventilated and were not sedated - to allow mechanical ventilation in the awake state, and to exclude the impact that sedative or narcotic drugs have on vascular tone. The unchanged blood flow in the sham group supports the ventilation-related decrease in PaCO_2 _and the corresponding increase in arterial pH having no influence on cerebral blood flow in this model, and the PaCO_2 _as well as arterial pH were similar between all groups. The increase in cerebral blood flow in control animals is most probably due to a loss of cerebral autoregulation during hypotensive, hyperdynamic shock states, and consecutive hypoxia, displayed by the significant drop in the PaO_2_/FiO_2 _ratio in the control group. The decrease in cardiac MDA and 3-NT levels in rhAPC-treated animals led to improved systemic hemodynamics within the limits of the cerebral autoregulation, thereby stabilizing cerebral blood flow. APC has also been shown to cross the blood-brain barrier [36] and to have neuroprotective effects in ischemic stroke models [37] and heat stroke models [38]. The origin of cerebral dysfunction in patients with sepsis is still unclear and may be related to increased intracranial pressure due to increased cerebral blood flow. Little is known, however, about the effects of rhAPC in this setting.

A limitation of the present study might be that regional microvascular blood flow, although correctly used as a term, is not identical to microvascular perfusion, as perfusion of vessels below 15 μm could not be evaluated.

In the present study, the total urine output in rhAPC-treated animals was significantly higher than in controls, suggesting increased renal perfusion. The fluid resuscitation in all investigated animals was adjusted hourly to maintain hematocrit and to prevent hemoconcentration or hemodilution. The fluid intake in controls was significantly less than in rhAPC animals because, based on an increased urine output, greater amounts of fluids had to be administered in rhAPC-treated sheep to maintain hematocrit. Even though there was no statistical difference in the fluid balance between the injured groups, however, some of the macrohemodynamic and microhemodynamic findings with rhAPC may be related to a slightly higher fluid balance in the treatment group. Further research on the effects of rhAPC on renal perfusion is necessary to draw final conclusions.

The perfect approach of how to ventilate and what FiO_2 _value to use remains a controversial discussion. One could argue that a FiO_2 _of 1.0 as used in our study could lead to hyperoxia-induced pulmonary injury. Murakami and colleagues, however, have shown that a FiO_2 _of 1.0 in this model is safe up to 48 hours [12]. In addition, Hauser and colleagues [39] and Barth and colleagues [40] could show that hyperoxia may have protective effects during the early and late phases of septic shock in a swine model, which may lead to future investigations.

## Conclusions

The present study is the first demonstrating a link between rhAPC and the reduction of cardiac MDA and 3-NT levels with improved global hemodynamics as well as attenuated changes in visceral and cerebral microvascular blood flow in ALI and septic shock. Future studies are necessary to further investigate the role of rhAPC on cardiovascular function and cerebral blood flow.

## Key messages

• rhAPC reduced cardiac MDA levels in septic shock.

• rhAPC reduced cardiac 3-NT levels in septic shock.

• rhAPC improved global hemodynamics in septic shock.

• rhAPC attenuated changes in microcirculation in septic shock.

## Abbreviations

ALI: acute lung injury; APC: activated protein C; BL: baseline; CO: cardiac output; COHb: carboxyhemoglobin; ELISA: enzyme-linked immunosorbent assay; FiO_2_: fraction of inspired oxygen; MAP: mean arterial pressure; MDA: malondialdehyde; NO: nitric oxide; NO_x_: total amount of nitric oxide metabolites; 3-NT: 3-nitrotyrosine; ONOO^-^, peroxynitrite; PaCO_2_: partial pressure of arterial carbon dioxide in the blood; rhAPC: recombinant human activated protein C.

## Competing interests

The authors declare that they have no competing interests. In the present study, some animals from a previous study [8] were used. The previous study, however, reported data for pulmonary injury and airway obstruction. The hemodynamic and microcirculatory data of the present study are original and have not been previously published.

## Authors' contributions

MOM and DMM designed and carried out the experiments, analyzed and interpreted the data, and drafted the manuscript and revised it critically for important intellectual content. JFF contributed grant support and study design, carried out experiments, and revised the manuscript critically for important intellectual content. DLT contributed grant support, study design and interpretation of the data. CS contributed grant support and data interpretation. MW, LK and EMH performed 3-NT and MDA measurements, collected and analyzed samples, and interpreted some data. YN and LDT performed the complicated surgeries, and collected and analyzed data. DNH contributed with grant support, experimental design and data interpretation. All authors read and approved the final manuscript, and decided on submission to *Critical Care*.
